# Stress Field Evaluation in Orthotropic Microstructured Composites with Holes as Cosserat Continuum

**DOI:** 10.3390/ma15186196

**Published:** 2022-09-06

**Authors:** Farui Shi, Nicholas Fantuzzi, Patrizia Trovalusci, Yong Li, Zuoan Wei

**Affiliations:** 1 State Key Laboratory of Coal Mine Disaster Dynamics and Control, Chongqing University, No. 174 Shazhengjie, Shapingba, Chongqing 400044, China; 2 School of Resources and Safety Engineering, Chongqing University, No. 174 Shazhengjie, Shapingba, Chongqing 400044, China; 3 Department of Civil, Chemical, Environmental and Materials Engineering, University of Bologna, Viale del Risorgimento 2, 40136 Bologna, Italy; 4 Department of Structural and Geotechnical Engineering, Sapienza University of Rome, Via A. Gramsci 53, 00197 Rome, Italy

**Keywords:** composite materials, microstructure direction, Cosserat continuum, stress concentration, scale effect

## Abstract

It is known that the presence of microstructures in solids such as joints and interfaces has an essential influence on the studies of the development of advanced materials, rock mechanics, civil engineering, and so on. However, microstructures are often neglected in the classical local (Cauchy) continuum model, resulting in inaccurate descriptions of the behavior of microstructured materials. In this work, in order to show the impact of microstructures, an implicit ‘non-local’ model, i.e., micropolar continuum (Cosserat), is used to numerically investigate the effects of direction and scale of microstructures on the tension problem of a composite plate with a circular hole. The results show that distributions of field variables (such as displacements and stresses) have an obvious directionality with respect to the microstructures’ direction. As the scale of microstructures increases, such a direction effect becomes more evident. Unlike the isotropic material where stress concentration occurs at the vertex of the hole and the stress concentration factor is close to 3, for the microstructured composite, the stress concentration can be observed at any location depending on the microstructures’ directions, and the concentration factor can exceed 3 to a maximum close to 9 as the increasing scale of microstructures. In addition, differences in the mechanical behavior between Cosserat and Cauchy models can be also observed; such differences are more evident for the material showing a pronounced orthotropic nature.

## 1. Introduction

Microstructure is one of the most critical factors that involves many kinds of materials such as rock, ceramic, alloy, human cortical bone, etc. [1,2,3,4]. As an internal structure, microstructure can play a crucial role in determining the gross behavior and mechanical response of materials [5]. However, the microstructures in materials distribute randomly with different lengths and directions, which complicates the understanding of the material’s response. In general, materials with microstructure have weaker strength than in intact materials [6,7]. Guo et al. [8] experimentally investigated the effect of bedding angle in phyllite under unloading confining pressures, where the rock bedding joints can be regarded as microstructures. They found that the rock shows different strengths as the bedding angle changes. Numerical research on the tunnel surrounding rocks with different inclination angles also shows a directional effect on the distributions of displacements and stress around the tunnel [9]. By reviewing indentation tests at the micron scale, Bauer et al. [10] demonstrate that an obvious length scale effect (i.e., non-locality) can be found when the material’s intrinsic length scales are comparable with the dimension of specimens. Therefore, it is of importance to describe the macroscopic response of these materials by considering the influences of the microstructures.

The existence of microstructure results in the heterogeneity characteristic of materials. There are various methods that can be used to model the behavior of microstructured materials. Discrete modeling with interactions of each constituent in materials is a good option because it can produce an accurate result; however, this approach is often computationally cumbersome [11,12,13]. Alternatively, homogenizing the heterogeneous material as an equivalent continuum could be an efficient approach because it is faster and takes less computational cost [14]. Nevertheless, the application of this approach depends on selections of the homogenization method and macroscopic continuum theory that need to reveal the presence of microstructures. As is known, the classical Cauchy continuum may have disadvantages in describing the gross behavior of microstructured materials since it lacks in internal length descriptions [15,16]. This calls for the application of the non-local continuum theory, as this approach can reveal the presence of internal lengths [17]. In the non-local theory, internal lengths can be represented by adding extra degrees of freedom or parameter as internal variables, corresponding to the so-called implicit and explicit non-local descriptions, respectively [18,19].

The Cosserat continuum theory is a widely used implicit non-local model to investigate the microstructured material’s behavior. After the completed mathematical foundations of the micropolar continuum was achieved, this theory became very popular (since the 70s). The Cosserat model introduces to each material point an extra degree of freedom, termed microrotation, which is different from the local rigid rotation (i.e., macrorotation). As a result, the stress and strain fields become asymmetric in this model, which is different from the classical Cauchy model with symmetric measurements. Moraes et al. [1] found that the asymmetrical property in the Cosserat model can be helpful to improve the description of the mechanical behavior of the materials such as rocks. The asymmetric strains also correspond to the relative rotation between the microrotation and macrorotation. Pau and Trovalusci [20] found that the relative rotation is significant in anisotropic materials, whereas it can be negligible in orthotetragonal materials where the internal length trends to vanish. With the advantage of keeping the memory of the microstructure, the Cosserat continuum was used to study many kinds of materials such as layered materials [21,22], fiber-reinforced materials [23,24], granular materials [25], and composites [26,27,28]. Using a homogenization process for the Cosserat continuum, Trovalusci and Masiani [29] numerically and experimentally studied the mechanical behavior of an inclined masonry structure in which microstructures (interfaces) show a different direction from the ordinary masonry structure. However, the results of this literature only showed the micropolar effect resulted from the Cosserat continuum but the effect of microstructures direction was not further discussed.

In this work, the effects of the microstructure’s direction and length scale in a composite are studied to extend the understanding of microstructured materials, such as advanced materials with various microstructured and layered rocks with inclined angles. The composite considered here is made of rectangular blocks interacting with each other through their elastic interfaces, and it is homogenized as a Cosserat continuum by an energetic-equivalence-based homogenization technique [30]. Thus, the characteristic of non-locality is involved in this study. Six directions and four length scales of the microstructure are investigated by the finite element method (FEM) for a tension problem of a composite plate with a circular hole. Therefore, this work also focuses on the stress concentration problem of microstructured materials. This problem has been widely reported in previous studies [31,32,33,34]. Holes in materials can induce stress concentration around it and hence reduce the mechanical properties [35]. However, a number of solutions have been carried out for holes in isotropic plates [36]. With the increasing research interests on materials, especially with microstructures, it is essential to gain a better understanding in modeling the mechanical behavior of these materials.

This paper is structured as follows. After the introduction section, Section 2 introduces the Cosserat theory and its FEM implementation. Section 3 presents the model, parameters, methods, etc. used in the simulations of the tension problem for a plate with a circular hole. In Section 4, numerical simulations are conducted and results of displacements, stresses, and relative rotation are shown. The stress distribution around the hole is discussed and the simulation results are analyzed in Section 5. In the end, conclusions and remarks are drawn in Section 6.

## 2. Cosserat Continuum and Its FEM Implementation

The Cosserat continuum is considered to be a multi-scale tool [37] that can be used to investigate the mechanical behavior of materials where the microstructures and, in particular, internal lengths, play a crucial role. As an implicit’ non-local continuum, it is equipped with additional degrees of freedom revealing the presence of microstructures. That is, for two-dimensional (2D) Cosserat media, each material point has two translation degrees of freedom $u_{1},u_{2}$ and an additional microrotation degree of freedom ω. The microrotation ω is an independent degree of freedom and it is different from the macro-rotation θ which is defined as the skew-symmetric part of the gradient of displacement. Thus, a peculiar measurement, the relative rotation θ−ω, can be defined in this continuum. A general displacement vector for the Cosserat material point can be expressed as:

(1) $$d^{⊤}=\left[\begin{array}{ccc} u_{1} & u_{2} & \omega \end{array}\right]=\left[\begin{array}{cc} u^{⊤} & \omega \end{array}\right]$$

where $u^{⊤}=\left[\begin{array}{cc} u_{1} & u_{2} \end{array}\right]$. Due to the introduction of ω, the tangential strains in the Cosserat model are not reciprocal, i.e., $\varepsilon_{12}\neq \varepsilon_{21}$, and the microcurvature component is introduced as an additional strain measure; therefore, the linear strain–displacement relation can be expressed as:

(2) $$\left[\begin{array}{c} \varepsilon \\ \chi \end{array}\right]=\left[\begin{array}{cc} L & M\\ 0 & \nabla \end{array}\right]\left[\begin{array}{c} u\\ \omega \end{array}\right]$$

where $\varepsilon^{⊤}=\left[\begin{array}{cccc} \varepsilon_{11} & \varepsilon_{22} & \varepsilon_{12} & \varepsilon_{21} \end{array}\right]$ contains the normal and tangential strains and $\chi^{⊤}=\left[\begin{array}{cc} \chi_{1} & \chi_{2} \end{array}\right]$ contains the microcurvatures. ∇ is the gradient operator, and

(3) $$\begin{array}{cc} L & ={\left[\begin{array}{cccc} \frac{\partial }{\partial x_{1}} & 0 & \frac{\partial }{\partial x_{2}} & 0\\ 0 & \frac{\partial }{\partial x_{2}} & 0 & \frac{\partial }{\partial x_{1}} \end{array}\right]}^{⊤},\\ M & ={\left[\begin{array}{cccc} 0 & 0 & 1 & -1 \end{array}\right]}^{⊤} \end{array}$$

With the strain measures, the stresses of the Cosserat continuum can be obtained by a linear elastic constitutive equation as:

(4) $$\left[\begin{array}{c} \sigma \\ \mu \end{array}\right]=\left[\begin{array}{cc} A & B\\ B^{⊤} & D \end{array}\right]\left[\begin{array}{c} \varepsilon \\ \chi \end{array}\right]$$

where $\sigma^{⊤}=\left[\begin{array}{cccc} \sigma_{11} & \sigma_{22} & \sigma_{12} & \sigma_{21} \end{array}\right]$ contains the normal and tangential stresses and $\mu^{⊤}=\left[\begin{array}{cc} \mu_{1} & \mu_{2} \end{array}\right]$ contains the couple stresses. The tangential stress components are also not reciprocal ($\sigma_{12}\neq \sigma_{21}$). The constitutive sub-matrices A, B, and D collect the constitutive terms $A_{ijhk},B_{ijh}$, and $D_{ij}$, where i,j,h and k=1,2.

A detailed three-dimensional finite element formulation for the Cosserat continuum can be found in [38]. For the sake of simplicity, the displacement-based finite element implementation for 2D Cosserat theory is presented here to model the behavior of microstructured material. Firstly, in the finite element procedure, displacement and microrotation fields should be approximated by the nodal values of an element. In this study, for avoiding the element locking problem, a bi-quadratic ($N_{u}$) and a bi-linear ($N_{\omega }$) shape function are, respectively, used for the displacement and microrotation approximation:

(5) $$\begin{array}{cc} u & =N_{u}\tilde{u}\\ \omega & =N_{\omega }\tilde{\omega } \end{array}$$

where $\tilde{u}$ and $\tilde{\omega }$ are nodal displacement and microrotation values. In the present paper, nine-node quadrangular elements are considered for an element. All nine node values are used to approximate the displacements, whereas values at four corner nodes are used for the microrotation. $N_{u}$ and $N_{\omega }$ can be expressed as:

(6) $$\begin{array}{cc} N_{u} & =\left[\begin{array}{ccccc} {N_{1}}^{u} & 0 & \cdots & {N_{9}}^{u} & 0\\ 0 & {N_{1}}^{u} & 0 & \cdots & {N_{9}}^{u} \end{array}\right],\\ N_{\omega } & =\left[\begin{array}{ccc} {N_{1}}^{\omega } & \cdots & {N_{4}}^{\omega } \end{array}\right] \end{array}$$

Substituting Equation (5) into (2), the strain vectors become:

(7) $$\begin{array}{cc} \varepsilon & =\left[\begin{array}{cc} {\mathrm{LN}}_{u} & {\mathrm{MN}}_{\omega } \end{array}\right]{\left\{\begin{array}{cc} \tilde{u} & \tilde{\omega } \end{array}\right\}}^{⊤}=B_{\varepsilon }\tilde{d},\\ \chi & =\left[\begin{array}{cc} 0 & \nabla N_{\omega } \end{array}\right]{\left\{\begin{array}{cc} \tilde{u} & \tilde{\omega } \end{array}\right\}}^{⊤}=B_{\chi }\tilde{d} \end{array}$$

where $B_{\varepsilon }$ and $B_{\chi }$ are the derivatives of the shape functions. $\tilde{d}$ is the unknown nodal values collecting $\tilde{u}$ and $\tilde{\omega }$. Substituting Equation (7) into Equation (4), the constitutive relations become:

(8) $$\begin{array}{cc} \sigma & =AB_{\varepsilon }\tilde{d}+BB_{\chi }\tilde{d},\\ \mu & =B^{⊤}B_{\varepsilon }\tilde{d}+DB_{\chi }\tilde{d} \end{array}$$

Now, the stress and couple stress measures can be obtained from the nodal values. Considering a domain A and boundary Γ, the principle of virtual work can be expressed as:

(9) $$\int_{A}\delta \varepsilon^{⊤}\sigma +\delta \chi^{⊤}\mu \;dA=\int_{A}\delta u^{⊤}b\;dA+\int_{\Gamma }\delta u^{⊤}\bar{t}+\delta \omega^{⊤}\bar{m}\;d\Gamma \;\forall \delta u,\delta \omega$$

where δ is the variational operator, b is the body force vector. $\bar{t}$ and $\bar{m}$ are the traction and couple-traction vectors applied on the boundary Γ. The components ($t_{i}$ and $m_{i}$) of $\bar{t}$ and $\bar{m}$ should satisfy the equilibrium at external boundary as $t_{i}=\sigma_{ij}n_{j}$ and $m_{i}=\mu_{j}n_{j}$, where $n_{j}$ is the components of the outward unit normal to the boundary. Substituting Equations (5), (7) and (8) into (9) and excluding body forces, we obtain:

(10) $$\delta {\tilde{d}}^{⊤}\underset{K^{e}}{\underset{︸}{\int_{A^{e}}\left(B_{\varepsilon }^{⊤}AB_{\varepsilon }+B_{\varepsilon }^{⊤}BB_{\chi }+B_{\chi }^{⊤}B^{⊤}B_{\varepsilon }+B_{\chi }^{⊤}DB_{\chi }\right)dA^{e}}}\;\tilde{d}=\delta {\tilde{d}}^{⊤}\underset{F^{e}}{\underset{︸}{\int_{\Gamma^{e}}\left[\begin{array}{c} N_{u}^{⊤}\bar{t}\\ N_{\omega }^{⊤}\bar{m} \end{array}\right]d\Gamma^{e}}}\;\forall \delta \tilde{d}$$

where $K^{e}$ and $F^{e}$ are the element stiffness matrix and the element nodal force vector. They can be computed numerically by a Gauss–Legendre integration with 3×3 grid. If considering arbitrary $\delta \tilde{d}$, we can obtain the standard finite element formulation as:

(11) $$K^{e}\tilde{d}=F^{e}$$

At the end, the unknown $\tilde{d}$ can be obtained by solving this equation. With this solution, in the post-processing stresses and strains are firstly computed at Gauss points for each element and then an extrapolation technique is used to get stresses and strains at element nodal points.

The above implementations are achieved by an updated MATLAB code based on codes of a classical 2D Cauchy continuum as presented in [39].

## 3. Numerical Simulation

**Figure 1:**
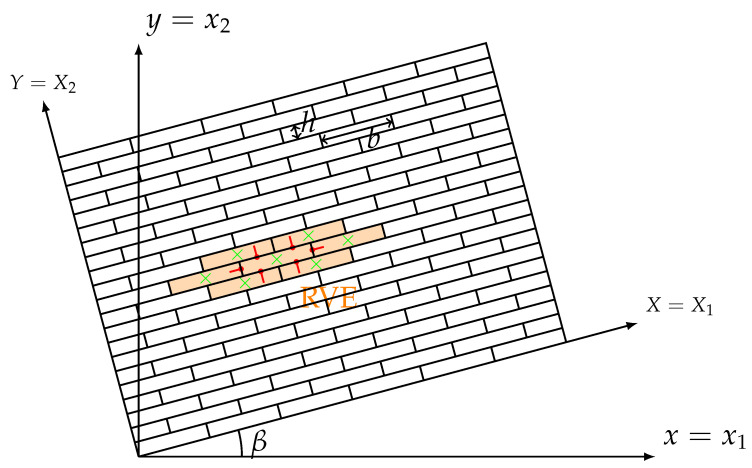
Schemes of the considered assembly and RVE.

In this section, we intend to numerically investigate the effects of direction and scale of microstructures for a composite material that can be considered as an assembly made of rigid rectangular blocks in contact with elastic interfaces (Figure 1), where each rectangular block has the width of *b* and height of *h*. The assembly is arranged as an interlocking structure and the interfaces of blocks form the microstructures of this composite material. A homogenization procedure presented in [30] can be used to describe the assembly as an equivalent Cosserat continuum. In this work, 7-block representative volume element (RVE) that is highlighted with orange color in Figure 1 is used for the homogenization procedure to produce the Cosserat constitutive matrix in Equation (4). In the highlighted RVE, the blocks’ centroids are represented by green crosses, and red lines mean the outward unit normal vectors of the central block’s interfaces. The direction of microstructures can be changed by transforming the assembly of an angle β from x−y coordinate system to X−Y coordinate system as shown in Figure 1. In this study, we select 6 values of β ($0^{\circ },30^{\circ },60^{\circ },90^{\circ },120^{\circ },150^{\circ }$). Furthermore, to obtain various scales of microstructures, 4 different block sizes are used by fixing the height of block h=0.1 m and changing the aspect ratio ρ=b/h=1.5,3,7, and 15, where a greater ρ corresponds to a longer rectangular block, as a consequence, showing more orthotropic nature.

The blocks interact among themselves through elastic common interfaces. The adopted spring stiffness at the interfaces is:

(12) $$K=\left[\begin{array}{cc} k_{n} & 0\\ 0 & k_{t} \end{array}\right]$$

where $k_{n}$ and $k_{t}$ are the normal and tangential stiffness per unit length, respectively. Here we have $k_{n}=576.58$ MPa/m and $k_{t}=288.29$ MPa/m. The rotation stiffness of interface is computed $k_{r}=k_{n}{\left(d/2\right)}^{2}$, where *d* is the length of interface. Therefore, the Cosserat constitutive matrices of the reference RVE when $\beta =0^{\circ }$ can be obtained by the homogenization technique that is based on an equivalence energy criterion between the material’s discrete system of and the continuum model [30]. The constitutive matrix of transformed assembly can be obtained as follows:

(13) $$C=Q^{⊤}C_{0}Q$$

where $C_{0}$ is the constitutive matrix when $\beta =0^{\circ }$, Q is the usual transformation matrix [40].

For comparison, the Cauchy continuum is also considered here to carry out the same simulations as performed by the Cosserat continuum. Because of the lack in microstructures, the constitutive relation of the Cauchy continuum has the form of: $\sigma =\hat{A}\varepsilon$, where the matrix $\hat{A}$ is obtained from A and their relationship can be found in the previous literature as [41]:

(14) $$\begin{array}{cc} {\hat{A}}_{1111} & =A_{1111}\\ {\hat{A}}_{1122} & =A_{1122}\\ {\hat{A}}_{2222} & =A_{2222}\\ {\hat{A}}_{1112} & =(A_{1112}+A_{1121})/2\\ {\hat{A}}_{2212} & =(A_{2212}+A_{2221})/2\\ {\hat{A}}_{1212} & =(A_{1212}+A_{2121}+2A_{1221})/4 \end{array}$$

Table 1, Table 2, Table 3 and Table 4 list the constitutive components of all configurations, where components keeping zero for all configurations are not reported. It can be seen that there are more zero components when $\beta =0^{\circ }$ and $90^{\circ }$. Actually, only diagonal components of the constitutive matrix exist and an orthotropic nature of the material is observed for these two transformation angles. For other angles, all components of matrices A and D appear. Consequently, materials with these RVEs show a centrosymmetric nature [30]. B=0 for all configurations, meaning there is no coupling between stresses/microcurvatures and microcouples/strains. As the aspect ratio ρ increases, constitutive components change monotonously except for $A_{2222}$, $A_{1212}$ of Cosserat continuum and ${\hat{A}}_{2222}$ of Cauchy continuum when $\beta =0^{\circ }$. Because of the fixed height of blocks (*h*), these components stay the same with increasing ρ.

**Figure 2:**
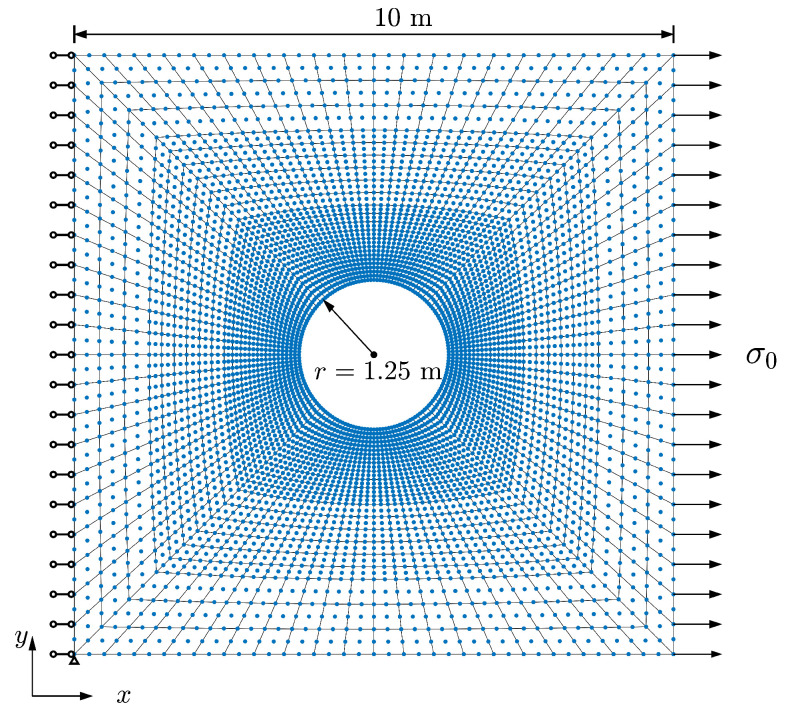
Sketch of the plate with hole problem and its finite element mesh.

In the following, a classical tension problem of a square plate with a circular hole is studied for all above-mentioned configurations. Figure 2 shows the sketch of the problem and its finite element meshing. The plate has a side length of 10 m and the radius of the hole is 1.25 m. A total of 1440 elements is used for this model. Due to the singular nature resulting from the presence of the hole, stress concentration is more likely observed around the hole under tension force. To make sure the results are reliable, a finer mesh is applied near the hole. Uniform tensile stress $\sigma_{0}=1$ MPa is applied on the right side of the plate. The left side of the plate is fixed symmetrically in the *x*-direction and the bottom left point is additionally fixed in the *y*-direction. In the present paper, the direction and scale effect of microstructure is investigated by setting various β and ρ. The simulation results are shown below.

## 4. Results

**Figure 3:**
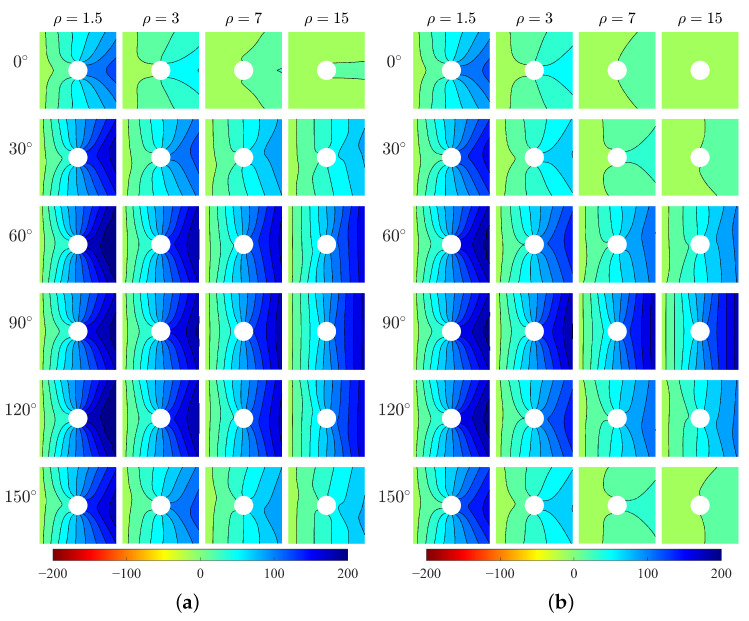
Horizontal displacement $u_{1}$, mm, (**a**) Cosserat, (**b**) Cauchy.

The results of displacements and stresses for the Cosserat and Cauchy models and relative rotation for the Cosserat model are presented in this section to show the effects of microstructure’s direction and scale on the behavior of microstructured composite materials. Figure 3 depicts the horizontal displacement results $u_{1}$ of Cosserat and Cauchy models. It can be seen that change in direction of microstructure has a significant effect on $u_{1}$ for both two models. The smallest $u_{1}$ can be observed when $\beta =0^{\circ }$. As β changes from $0^{\circ }$ to $150^{\circ }$, $u_{1}$ increases to the greatest at $\beta =90^{\circ }$ and then decreases but this is expected for the shortest block case ρ=1.5. In the case of ρ=1.5, the greatest $u_{1}$ occurs at $\beta =60^{\circ }$ and $120^{\circ }$ but that is also close to $u_{1}$ at $\beta =90^{\circ }$. For all β representing the directions of microstructure, $u_{1}$ has a reduction as ρ increases. For the plate with the shortest blocks, displacement localization can be observed at the middle of the right side of the plate. However, as ρ increases, displacement localization reduces and uniform displacement distribution can be observed at the right side of the plate.

It can be seen from Figure 3 that the difference in $u_{1}$ between Cosserat and Cauchy models is not obvious for the orthotropic materials ($\beta =0^{\circ }$ and $90^{\circ }$). Here we take the difference in the maximum $u_{1}$ between the two models as $\Delta u_{1}$. For various ρ, $\Delta u_{1}$ is 3.02–8.63 mm when $\beta =0^{\circ }$ and 2.13–5.85 mm when $\beta =90^{\circ }$. However, the difference is more evident for the centrosymmetric materials. $\Delta u_{1}$ increases from 20 mm to 69 mm as ρ increases when $\beta =30^{\circ },60^{\circ },120^{\circ }$, and $150^{\circ }$.

**Figure 4:**
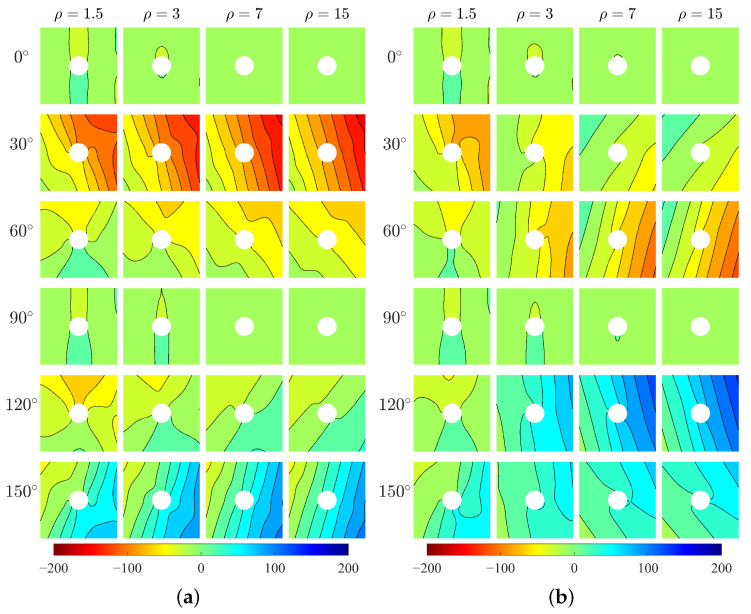
Vertical displacement $u_{2}$, mm, (**a**) Cosserat, (**b**) Cauchy.

Figure 4 depicts the vertical displacement results $u_{2}$ for two models. The orthotropic materials ($\beta =0^{\circ }$ and $90^{\circ }$) show negligible $u_{2}$ under the horizontal tension stress for both models. However, the centrosymmetric materials with other transformation angles can produce clear $u_{2}$ with more or less directionality.

It should be noted that there is a big difference in $u_{2}$ between Cosserat and Cauchy models. $u_{2}$ of these two models has a similar distribution only when ρ=1.5. As ρ increases, $u_{2}$ distributions of two models become different. When $\beta =30^{\circ }$ and $150^{\circ }$, the Cosserat continuum shows high-intensity $u_{2}$ distribution on the right plate as ρ increases, e.g., the maximum $u_{2}$ is up to 117 mm when $\beta =150^{\circ }$; however, $u_{2}$ from the Cauchy continuum has no clear high-intensity distribution with ρ and the maximum $u_{2}$ is just 77 mm when $\beta =150^{\circ }$. An opposite difference between two models can be observed when $\beta =60^{\circ }$ and $120^{\circ }$, that is high-intensity $u_{2}$ distribution can be observed by the Cauchy but not the Cosserat model.

**Figure 5:**
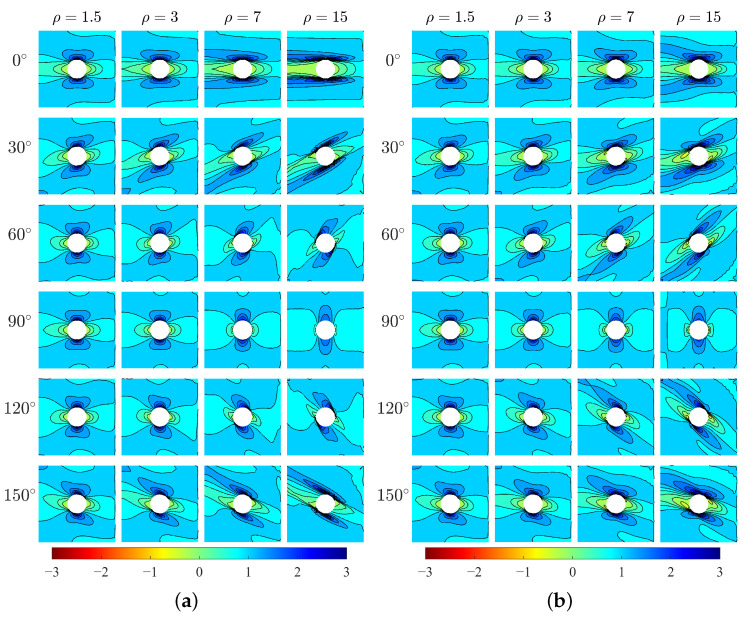
Horizontal stress $\sigma_{11}$, MPa, (**a**) Cosserat, (**b**) Cauchy.

The horizontal stresses $\sigma_{11}$ of the two models are shown in Figure 5. The results show directionality of distribution of $\sigma_{11}$ with respect to the direction β. For orthotropic materials, the high-stress area is parallel to the *x*-direction and the peak stress happens at the top and bottom points of the hole edge (i.e., x=0). However, for centrosymmetric materials, the high-stress area is inclined with the *x*-direction by an angle that can be related to the direction of microstructure, and the location of peak stress changes. It can be seen the directionality of $\sigma_{11}$ is more evident for greater ρ. As the increase of ρ, the high-stress area becomes wider. The exception happens at $\beta =90^{\circ }$ which shows the opposite behavior. The existence of the hole as a singularity can result in the concentration of stress around the hole edge. In the following, the stress concentration at the hole boundary will be discussed.

**Figure 6:**
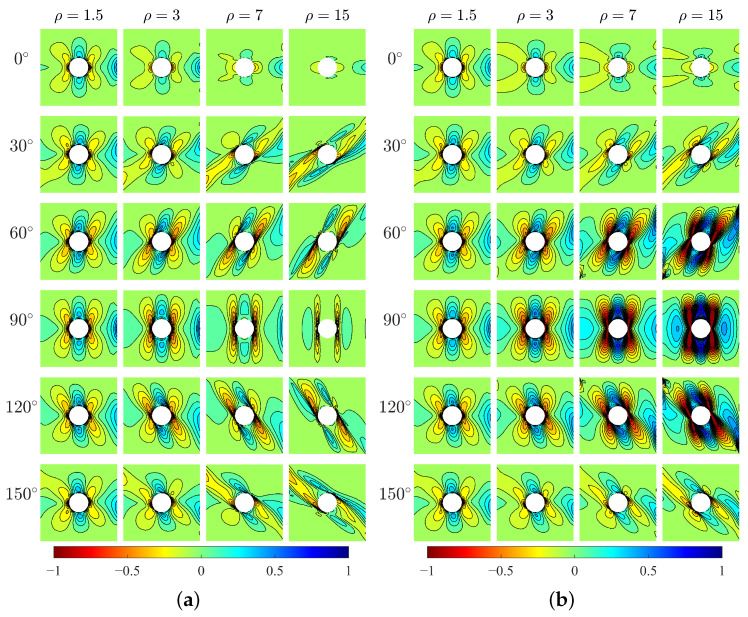
Vertical stress $\sigma_{22}$, MPa, (**a**) Cosserat, (**b**) Cauchy.

Vertical stress $\sigma_{22}$ (Figure 6) also shows directionality of stress distribution with respect to β. There can be seen a difference in $\sigma_{22}$ between two models for various ρ. For relative short blocks (ρ=1.5 and 3), two models produce closed behavior of $\sigma_{22}$. However, for longer blocks (ρ=7 and 15) especially at $\beta =60^{\circ },90^{\circ }$ and $120^{\circ }$, $\sigma_{22}$ from Cauchy model is significantly greater than that from Cosserat model.

**Figure 7:**
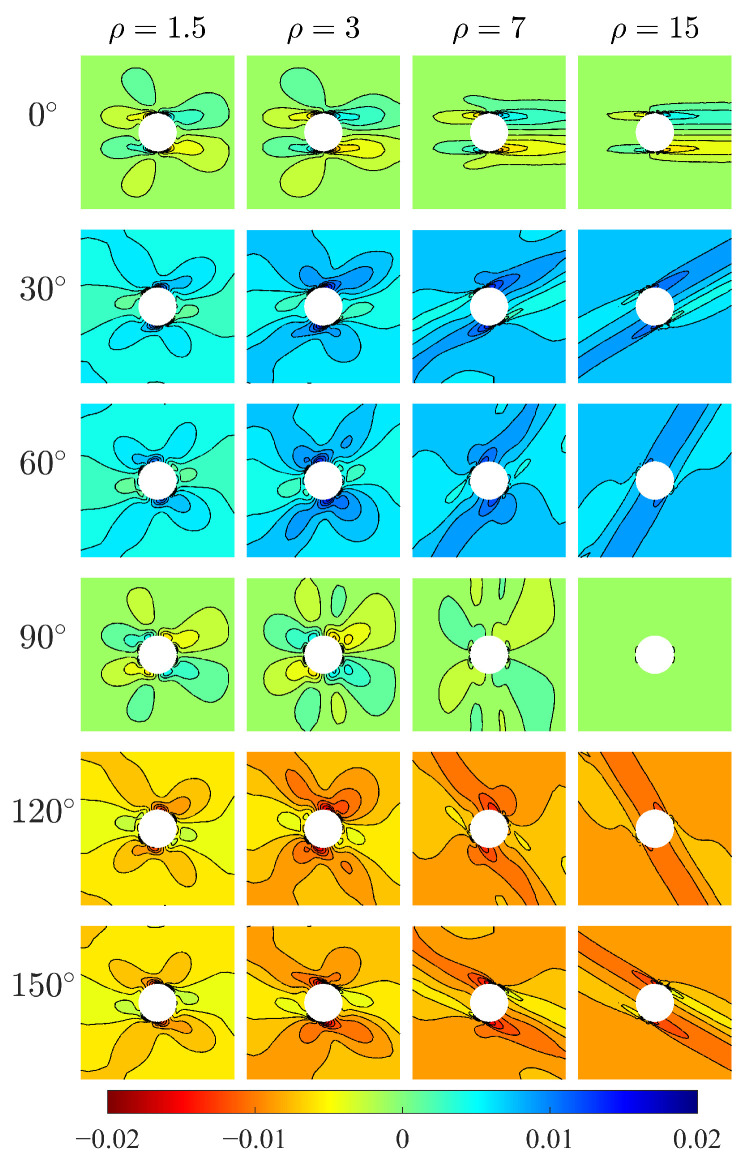
Relative rotation θ−ω of Cosserat model.

The relative rotation, defined as the difference between macrorotation (θ) and microrotation (ω), is a peculiar measurement in the Cosserat model. Figure 7 shows the relative rotation θ−ω for the Cosserat model. The directionality of θ−ω distribution can be also observed and that is more evident as ρ increases. For orthotropic materials, θ−ω shows point symmetric behavior with respect to the hole center where positive and negative θ−ω can be both observed, but the value of θ−ω is close to 0. Thus, the Cosserat model is very close to the Cauchy one. For $\beta =30^{\circ }$ and $60^{\circ }$, the plate domain mainly undergoes a positive relative rotation. Oppositely, for $\beta =120^{\circ }$ and $150^{\circ }$, this domain mainly undergoes a negative θ−ω. The above-mentioned indicates that relative rotation can be affected by the direction of microstructure. There is less relative rotation when the microstructures are arranged along parallel and perpendicular to the direction of force, whereas the microstructures arranged along other directions would result in an obvious relative rotation acting at a certain orientation.

## 5. Discussions

**Figure 8:**
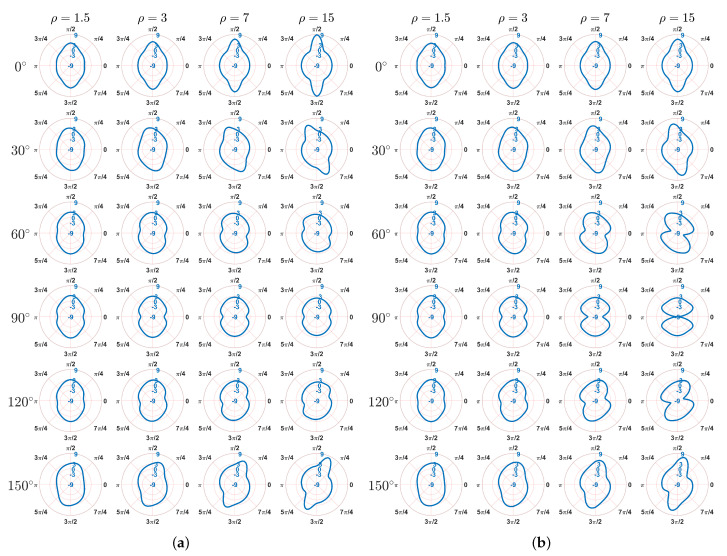
Distribution of the hoop stress $\sigma_{h}$ at the hole boundary, MPa, (**a**) Cosserat, (**b**) Cauchy.

The problem of stress concentration has always been focused on due to the presence of singularity [32,42,43]. Under horizontal tension in this study, it can be seen that the stresses $\sigma_{11}$ and $\sigma_{22}$ in the plate (Figure 5 and Figure 6) is mainly concentrated around the boundary of the hole. To better show the stress distribution, in the following, by transforming the stress state from Cartesian coordinate to polar coordinate, the hoop stress $\sigma_{h}$ at the hole boundary is depicted for two models in the polar coordinate system as shown in Figure 8. In this way, $\sigma_{h}$ represents $\sigma_{22}$ when the polar angle equals to 0 or π, whereas the hoop stress denotes $\sigma_{11}$ when the polar angle is π/2 or 3π/2. Therefore, the location and magnitude of $\sigma_{h}$ can be clearly observed. For both Cosserat and Cauchy models, it can be seen that the distribution of $\sigma_{h}$ at the hole boundary is point symmetric to the center of hole and also shows directionality that depends on the aspect ration ρ and angle β.

For the orthotropic materials, the distribution of $\sigma_{h}$ is symmetrical along the vertical direction (π/2−3π/2) in the polar coordinate. The highest $\sigma_{h}$ can be observed at the top and bottom points of the hole boundary (i.e., polar angle equals to π/2 and 3π/2) for the Cosserat model and Cauchy model when $\beta =0^{\circ }$, indicating that the peak stress results from the horizontal stress $\sigma_{11}$. As for the Cauchy model when $\beta =90^{\circ }$, it is consistent with the above results for small ρ. As ρ increases, the highest $\sigma_{h}$ trend to be occurred at the right and left points of the hole boundary (i.e., polar angle equals to 0 and π). Since we observed a significant $\sigma_{22}$ concentration in the Cauchy model in Figure 6b, the vertical stress $\sigma_{22}$ is able to result in the peak stress for these cases. For centrosymmetric materials, the distribution of $\sigma_{h}$ is no longer symmetrical along the vertical direction but deviates from it to more or less of an extent because of the transformation angle of rectangular blocks. Thus, the highest $\sigma_{h}$ does not occur at these special points, i.e., polar angle equals to 0,π/2,π, or 3π/2. This is consistent with the results by early study [9], which investigates the stress distribution of the layered surrounding rock tunnel by considering different angles of rock joints (microstructures). It is also shown that the stress distribution is symmetrical when angle equals to $0^{\circ }$ and $90^{\circ }$. When the angle is $45^{\circ }$ the stress presents an asymmetric distribution and the tunnel even comes into being eccentric-pressed.

It should be noted that the effect of β is less for smaller ρ. When ρ=1.5, the distribution of $\sigma_{h}$ is close to each other, that is, the highest $\sigma_{h}$ is located near to the top and bottom point of hole boundary and its value close to 3, whereas the lowest $\sigma_{h}$ near to right and left points and its value is around 1. Such a result is close to the well-known analytical solution for an infinite isotropic plate with a circular hole [33,44]. However, as ρ increases, the directionality of $\sigma_{h}$ distribution becomes more evident and the extreme values of $\sigma_{h}$ also vary. For example, when $\beta =0^{\circ }$, as increase in ρ the highest value of $\sigma_{h}$ can increase to 9. In addition, the difference between the Cosserat and Cauchy models gets bigger with increasing ρ. The smallest values of $\sigma_{h}$ are not lower than −3 for the Cosserat continuum, whereas lower $\sigma_{h}$ to −9 can be obtained from the Cauchy continuum, especially when $\beta =60^{\circ },90^{\circ }$, and $120^{\circ }$.

By investigating measurements of displacements, stress, etc, and the stress distribution of the square plate with circular hole under horizontal tension stress, a direction effect of microstructures can be found for the present problem. With different transformation angle β, the plate can produce mechanical behavior showing obvious directionality. Such a direction effect of microstructures can be also found in previous studies on surrounding rock roadway and tunnels with different dip angles of surroundings [9,45], where the joints of surrounding rocks can represent the microstructure interfaces. In this present paper, similar behaviors are often observed when $\beta =0^{\circ }$ and $90^{\circ }$. That is because the microstructures in these two cases are both parallel and perpendicular to the *x*-direction, showing an orthotropic nature. The difference between these two cases is actually due to the different values of ρ (ρ<1 when $\beta =90^{\circ }$ whereas ρ>1 when $\beta =0^{\circ }$); therefore, measurements of these two cases have similar behavior but different intensities under the horizontal tension stress. As for microstructures not parallel or perpendicular to the *x*-direction, i.e., $\beta =30^{\circ },60^{\circ },120^{\circ }$ and $150^{\circ }$, it can be seen from the constitutive matrices (Table 1, Table 2, Table 3 and Table 4) that more coupling in the constitutive components such as dilatancy components [46] appear for these cases, showing a centrosymmetric nature. The centrosymmetric material can show different behavior from the orthotropic material by coupling different stresses and strains as well as the couple stresses and curvatures. As the transformed constitutive matrix is related to β (Equation (13)), the behavior of the centrosymmetric material depends on β. Under horizontal tension stress, it is shown that $u_{1}$ first increases and then decreases with β. Oppositely, as β increases the maximum $\sigma_{h}$ first decreases and then increases. Such an effect of β can be compared with the previous study [8] where a similar effect was found, that is, the strength of phyllite decreases first and then increases with the increase of bedding angle.

The direction effect of microstructures is more evident for higher ρ. When ρ is small (e.g., ρ=1.5), the width of the rectangular block *b* is close to its height *h*. Thus, the assembly made of such blocks can show a nearly orthotetragonal behavior (close to isotropic) that is less sensitive to change in the microstructure direction. This could also account for the small differences between Cosserat and Cauchy results when ρ is small since it was known that orthotetragonal materials are very close to Cauchy continua [30,47].

With the increase in ρ, the assembly becomes more orthotropic and the measurements can show more obvious directionality with respect to β. As the length of microstructure (i.e., internal length) is introduced to the Cosserat continuum, asymmetries are generated for the shear stress and shear strain fields. Therefore, in the Cosserat model each stress is coupled with asymmetric shear strains through two constitutive components ($A_{ij12}$ and $A_{ij21}$), and vice-versa. In the Cauchy model, there is just one component (${\hat{A}}_{ij12}$). When ρ is small, the difference between $A_{ij12}$ and $A_{ij21}$ is not evident. As ρ increases, such difference increases rapidly, showing a higher degree of asymmetry. However, ${\hat{A}}_{ij12}$ of the Cauchy model is an arithmetic mean by $A_{ij12}$ and $A_{ij21}$ (Equation (14)), which cannot show the asymmetric behavior of continua. Thus, the difference between the results from Cosserat and Cauchy models may be induced, especially for higher ρ. Moreover, since the microstructures are considered in the Cosserat continuum, the additional sub-matrix D is involved in the Cosserat constitutive relation rather than the Cauchy one. The components of D are negligible for small ρ but become prominent as ρ increases. It has been found that the stress can be re-distributed within the Cosserat continuum [34]. For the smaller ρ, the Cosserat continuum behaves close to the Cauchy continuum as mentioned above. As a result, the re-distribution of stress can be neglected. However, as the scale of the microstructure increases, such a re-distribution can be more prominent for higher ρ. This can be used to account for the difference in $\sigma_{22}$ between the Cosserat and Cauchy continuum, especially for large ρ.

## 6. Conclusions

The present paper investigates the mechanical behavior of the microstructured composite treated as Cosserat continuum by considering various microstructure’s directions (β) and scales (ρ). According to the constitutive parameters obtained from the Cosserat homogenization procedure, the composite studied here can be classified as orthotropic and centrosymmetric materials depending on the direction β. The simulations are conducted for a tension problem of a microstructured plate with a circular hole, so this paper also focuses on the stress distribution around the hole. The main conclusions are as follows:

(1) The mechanical behavior of microstructured composite changes as the microstructure’s directions β, thereby showing a directionality of measurement distribution such as stresses. In general, orthotropic materials show similar behaviors but with different intensities, and the behavior of centrosymmetric is related to various β.
(2) The increasing microstructure’s scale ρ can results in more evident effect of β and difference between the Cosserat and Cauchy models. Such an effect of ρ is clearer for the centrosymmetric materials than orthotropic materials.
(3) The Cosserat continuum is able to better describe the direction effect of microstructures due to the relative rotation that not only shows the directionality of distribution but also varies with the microstructure direction. The Cauchy continuum does not have such advantages because there is no relative rotation and tangential strains are symmetric.
(4) The extreme value and its location of the hoop stress $\sigma_{h}$ around the hole depend on β. For smaller ρ, the highest and smallest $\sigma_{h}$ are close to 3 and −1, which is similar with the classical result of the isotropic material. As ρ increases, a highest $\sigma_{h}$ up to 9 can be observed.
(5) Difference in the hoop stress $\sigma_{h}$ between the Cosserat and Cauchy model is mainly in the smallest $\sigma_{h}$, especially for greater ρ when $\beta =60^{\circ },90^{\circ }$, and $120^{\circ }$. All the smallest $\sigma_{h}$ of the Cosserat model are greater than -3, whereas the Cauchy model can have a $\sigma_{h}$ as low as −9.

From this present study, the effect of the microstructure’s directions on mechanical behavior of microstructured composite can be found, especially for the composite with large scale of the microstructure. The area applying the development of this research can be for microstructured materials with various dimensions (from micromaterials to macromaterials), where the scale of the microstructure should be comparable to the material’s dimension. For example, the layered rock with joints has different dip angle due to the geological formation. The stress distribution can be also used for such composite with singularity, not only the circular shape used here, but alternative shapes. The rectangular microstructures with standard interlocking give a basic and important research aspect of the effect of the microstructure’s direction and scale. As more and more advanced materials are developed nowadays, various microstructures formations are interesting to be studied in future research.

## Figures and Tables

**Table 1:** Cosserat and Cauchy constitutive parameters for RVE with block parameter ρ=1.5, $A_{ijkl},{\hat{A}}_{ijkl}$ [MPa], $D_{ij}$ [MPa·m${}^{2}$].

	$0^{\circ }$	$30^{\circ }$	$60^{\circ }$	$90^{\circ }$	$120^{\circ }$	$150^{\circ }$
$A_{1111}$	102.70	80.97	58.45	57.66	58.45	80.97
$A_{1122}$	0	10.47	10.47	0	10.47	10.47
$A_{1112}$	0	25.94	13.85	0	−13.85	−25.94
$A_{1121}$	0	5.66	−6.44	0	6.44	−5.66
$A_{2222}$	57.66	58.45	80.97	102.70	80.97	58.45
$A_{2212}$	0	−6.44	5.66	0	−5.66	6.44
$A_{2221}$	0	13.85	25.94	0	−25.94	−13.85
$A_{1212}$	28.83	51.01	74.44	75.68	74.44	51.01
$A_{1221}$	0	10.47	10.47	0	10.47	10.47
$A_{2121}$	75.68	74.44	51.01	28.83	51.01	74.44
$D_{11}$	0.57	0.47	0.29	0.19	0.29	0.47
$D_{12}$	0	0.16	0.16	0	−0.16	−0.16
$D_{22}$	0.19	0.29	0.47	0.57	0.47	0.29
${\hat{A}}_{1111}$	102.70	80.97	58.45	57.66	58.45	80.97
${\hat{A}}_{1122}$	0	10.47	10.47	0	10.47	10.47
${\hat{A}}_{1112}$	0	15.80	3.71	0	−3.71	−15.80
${\hat{A}}_{2222}$	57.66	58.45	80.97	102.70	80.97	58.45
${\hat{A}}_{2212}$	0	3.71	15.80	0	−15.80	−3.71
${\hat{A}}_{1212}$	26.13	36.60	36.60	26.13	36.60	36.60

**Table 2:** Cosserat and Cauchy constitutive parameters for RVE with block parameter ρ=3, $A_{ijkl},{\hat{A}}_{ijkl}$ [MPa], $D_{ij}$ [MPa·m${}^{2}$].

	$0^{\circ }$	$30^{\circ }$	$60^{\circ }$	$90^{\circ }$	$120^{\circ }$	$150^{\circ }$
$A_{1111}$	237.84	183.34	93.24	57.66	93.24	183.34
$A_{1122}$	0	9.46	9.46	0	9.46	9.46
$A_{1112}$	0	85.04	74.12	0	−74.12	−85.04
$A_{1121}$	0	3.90	−7.02	0	7.02	−3.90
$A_{2222}$	57.66	93.24	183.34	237.84	183.34	93.24
$A_{2212}$	0	−7.02	3.90	0	−3.90	7.02
$A_{2221}$	0	74.12	85.04	0	−85.04	−74.12
$A_{1212}$	28.83	85.14	178.83	216.22	178.83	85.14
$A_{1221}$	0	9.46	9.46	0	9.46	9.46
$A_{2121}$	216.22	178.83	85.14	28.83	85.14	178.83
$D_{11}$	3.64	2.87	1.33	0.56	1.33	2.87
$D_{12}$	0	1.33	1.33	0	−1.33	−1.33
$D_{22}$	0.56	1.33	2.87	3.64	2.87	1.33
${\hat{A}}_{1111}$	237.84	183.34	93.24	57.66	93.24	183.34
${\hat{A}}_{1122}$	0	9.46	9.46	0	9.46	9.46
${\hat{A}}_{1112}$	0	44.47	33.55	0	−33.55	−44.47
${\hat{A}}_{2222}$	57.66	93.24	183.34	237.84	183.34	93.24
${\hat{A}}_{2212}$	0	33.55	44.47	0	−44.47	−33.55
${\hat{A}}_{1212}$	61.26	70.72	70.72	61.26	70.72	70.72

**Table 3:** Cosserat and Cauchy constitutive parameters for RVE with block parameter ρ=7, $A_{ijkl},{\hat{A}}_{ijkl}$ [MPa], $D_{ij}$ [MPa·m${}^{2}$].

	$0^{\circ }$	$30^{\circ }$	$60^{\circ }$	$90^{\circ }$	$120^{\circ }$	$150^{\circ }$
$A_{1111}$	756.76	604.96	255.41	57.66	255.41	604.96
$A_{1122}$	0	−22.97	−22.97	0	−22.97	−22.97
$A_{1112}$	0	328.47	354.99	0	−354.99	−328.47
$A_{1121}$	0	−52.27	−25.75	0	25.75	52.27
$A_{2222}$	57.66	255.41	604.96	756.76	604.96	255.41
$A_{2212}$	0	−25.75	−52.27	0	52.27	25.75
$A_{2221}$	0	354.99	328.47	0	−328.47	−354.99
$A_{1212}$	28.83	225.68	665.32	908.11	665.32	225.68
$A_{1221}$	0	−22.97	−22.97	0	−22.97	−22.97
$A_{2121}$	908.11	665.32	225.68	28.83	225.68	665.32
$D_{11}$	59.06	44.97	16.81	2.72	16.81	44.97
$D_{12}$	0	24.40	24.40	0	−24.40	−24.40
$D_{22}$	2.72	16.81	44.97	59.06	44.97	16.81
${\hat{A}}_{1111}$	756.76	604.96	255.41	57.66	255.41	604.96
${\hat{A}}_{1122}$	0	−22.97	−22.97	0	−22.97	−22.97
${\hat{A}}_{1112}$	0	138.10	164.62	0	−164.62	−138.10
${\hat{A}}_{2222}$	57.66	255.41	604.96	756.76	604.96	255.41
${\hat{A}}_{2212}$	0	164.62	138.10	0	−138.10	−164.62
${\hat{A}}_{1212}$	234.23	211.26	211.26	234.23	211.26	211.26

**Table 4:** Cosserat and Cauchy constitutive parameters for RVE with block parameter ρ=15, $A_{ijkl},{\hat{A}}_{ijkl}$ [MPa], $D_{ij}$ [MPa·m${}^{2}$].

	$0^{\circ }$	$30^{\circ }$	$60^{\circ }$	$90^{\circ }$	$120^{\circ }$	$150^{\circ }$
$A_{1111}$	2486.50	2096.86	882.44	57.66	882.44	2096.86
$A_{1122}$	0	−217.57	−217.57	0	−217.57	−217.57
$A_{1112}$	0	1189.82	1441.04	0	−1441.04	−1189.82
$A_{1121}$	0	−389.32	−138.10	0	138.10	389.32
$A_{2222}$	57.66	882.44	2096.86	2486.50	2096.86	882.44
$A_{2212}$	0	−138.10	−389.32	0	389.32	138.10
$A_{2221}$	0	1441.04	1189.82	0	−1189.82	−1441.04
$A_{1212}$	28.83	722.98	2546.41	3675.70	2546.41	722.98
$A_{1221}$	0	−217.57	−217.57	0	−217.57	−217.57
$A_{2121}$	3675.70	2546.41	722.98	28.83	722.98	2546.41
$D_{11}$	933.59	703.25	242.57	12.23	242.57	703.25
$D_{12}$	0	398.96	398.96	0	−398.96	−398.96
$D_{22}$	12.23	242.57	703.25	933.59	703.25	242.57
${\hat{A}}_{1111}$	2486.50	2096.86	882.44	57.66	882.44	2096.86
${\hat{A}}_{1122}$	0	−217.57	−217.57	0	−217.57	−217.57
${\hat{A}}_{1112}$	0	400.25	651.47	0	−651.47	−400.25
${\hat{A}}_{2222}$	57.66	882.44	2096.86	2486.50	2096.86	882.44
${\hat{A}}_{2212}$	0	651.47	400.25	0	−400.25	−651.47
${\hat{A}}_{1212}$	926.13	708.56	708.56	926.13	708.56	708.56

## Data Availability

Some or all data, models, or code that support the findings of this study are available from the corresponding author upon reasonable request.
